# Clinical Features of Ischemic Enteritis Diagnosed by Double-Balloon Endoscopy

**DOI:** 10.1155/2021/8875564

**Published:** 2021-04-14

**Authors:** Masanao Nakamura, Takeshi Yamamura, Keiko Maeda, Tsunaki Sawada, Yasuyuki Mizutani, Eri Ishikawa, Naomi Kakushima, Kazuhiro Furukawa, Takuya Ishikawa, Eizaburo Ohno, Takashi Honda, Hiroki Kawashima, Masatoshi Ishigami, Mitsuhiro Fujishiro

**Affiliations:** ^1^Department of Gastroenterology and Hepatology, Nagoya University Graduate School of Medicine, 65 Tsurumai-cho, Showa-ku, Nagoya 466-8550, Japan; ^2^Department of Endoscopy, Nagoya University Graduate School of Medicine, 65 Tsurumai-cho, Showa-ku, Nagoya 466-8550, Japan

## Abstract

**Introduction:**

Ischemic enteritis (IE) is a relatively rare small bowel disease that is diagnosed via double-balloon endoscopy (DBE), although the lack of established diagnostic criteria can make it difficult to confirm the diagnosis. This study aimed to describe the clinical characteristics, endoscopic imaging features, and treatments for IE at our center. *Patients and Methods*. We retrospectively searched the DBE database (1,521 patients) at Nagoya University Hospital for patients with IE and collected data regarding endoscopic findings, clinical background, and histological findings. The cases were categorized according to whether they involved transient or stenotic IE.

**Results:**

The DBE database included 24 patients (14 men) with IE. Transient IE was identified in 9 patients, and stenotic IE was identified in 15. Half of the patients had a history of cerebrovascular and cardiovascular disease. A granular structure at the ulcer base was the most frequently observed DBE finding at the stenotic site. Enterography using the contrast medium revealed that transient IE had a similar stenotic lesion length, relative to stenotic IE, although stenotic IE had a significantly higher stenosis ratio (81% vs. 63%, *P*=0.033). Small bowel enteroclysis revealed the “lead pipe” sign (11 patients), thumbprinting (3 patients), and the serrated lumen sign (1 patient). Only 1 patient with stenotic IE experienced recurrence after conservative treatment.

**Conclusion:**

During DBE, IE was characterized by cannular stenosis with extended and variable ulceration types, which spread over the edge of the stenosis, and a granular appearance at the ulcer base. These findings may help guide the diagnosis of IE.

## 1. Introduction

The small bowel has traditionally been considered the most difficult part of the gastrointestinal tract to access using endoscopic devices. However, the recent introduction of balloon-assisted endoscopy techniques, including double-balloon endoscopy (DBE), has allowed for endoscopic examination of the entire small bowel [1]. These techniques have also guided the observation and diagnosis of various small bowel lesions, although unidentified small bowel ulcers or stenosis are still encountered during DBE [2]. It can be difficult to diagnose these lesions, even in cases with endoscopic findings or histological results from a biopsy specimen [3].

Ischemic enteritis- (IE-) like lesions are often discovered by DBE, but definitive diagnostic criteria have not yet been established. For example, Raf reported that IE was only diagnosed in 11 among 9536 cases of surgery for nonmalignant small bowel disorders [4]. In the lower gastrointestinal field, ischemic colitis is more common than IE, as the small bowel is considered as an organ that has a rich collateral circulation [5]. IE can be classified as transient or stenotic IE according to the ischemic colitis status [5]. Transient IE involves small bowel stenosis that resolves to its original state after an acute onset, which is related to relatively mild ischemic changes. Stenotic IE involves stenosis that develops after chronic inflammation that is related to ischemia spreading from the submucosa to the muscularis propria. We are only aware of sporadic case reports of IE, which have generally described stenotic IE and provided endoscopic images [6–9]. Another report has described successful endoscopic balloon dilation for stenotic IE [10]. However, the clinical characteristics of IE are not fully understood [11, 12]. Therefore, we retrospectively evaluated the clinical characteristics, endoscopic findings, and treatments for IE using our DBE database.

### 1.1. Patients and Methods

This study's retrospective protocol was approved by the ethics committee of Nagoya University Hospital (2015–0485). Our clinical practice for diagnosing small bowel disease using DBE has been previously described [13, 14]. We usually complete a detailed evaluation by DBE for all cases of suspected IE. Since transient IE may heal naturally by fasting, DBE would be recommended early after onset.

Peroral and/or transanal routes are selected according to the suspected lesion's location, which is determined using the results of a clinical examination, computed tomography (CT) scan, and small bowel barium series. In DBE, total intestinal examination was usually completed when the entire small intestine was evaluated by both oral and anal approaches through DBE observation and fluoroscopy, with reference to specific landmarks, such as characteristic ulcers and stenosis [15].

We have previously defined the clinical, radiographical, and histopathological diagnostic criteria for IE according to published reports [11]. The etiology of small bowel IE is generally classified as extensive or segmental ischemia [5].

The DBE database was searched to identify demographic characteristics, endoscopic findings, and treatments for patients with IE. The ratio of stenosis based on the fluoroscopic DBE images was calculated according to the formula, 2 × (minimum diameter of short axis in the lesion site)/((short axis diameter of the normal intestinal cavity on the oral side nearby the lesion) + (short axis diameter of the normal intestinal cavity on the anal side nearby the lesion)) × 100%. Transient IE was defined as lesions that were improved by conservative treatment, while stenotic IE was defined as lesions that required invasive treatment (excluding endoscopic hemostatic procedures).

### 1.2. Statistical Analysis

The statistical analyses were performed using SPSS (version 26 for Windows; IBM Corp., Armonk, NY, USA) and Stat View software (SAS Inc., Cary, NC, USA). Comparisons between the groups were performed using the Mann–Whitney *U* test, Fisher's exact test, or the *χ*^2^ test, as appropriate. Differences were considered statistically significant at *P* values of <0.05.

## 2. Results

The DBE database at Nagoya University Hospital included 1521 patients who had undergone 2289 DBEs between June 2003 and December 2015. A total of 24 patients were diagnosed with IE, based on fulfilment of criteria 1 and 3 (11 patients) or criteria 2 and 3 (13 patients) (Table 1). The clinical characteristics of the patients with IE are given in Table 2. All patients were referred to our hospital from other hospitals and frequently had cerebrovascular or cardiovascular diseases, including diabetic microangiopathy. Nineteen patients had developed symptoms of progressive small bowel obstruction at the referring hospital.

### 2.1. CT Findings before DBE

**Figure 1:**
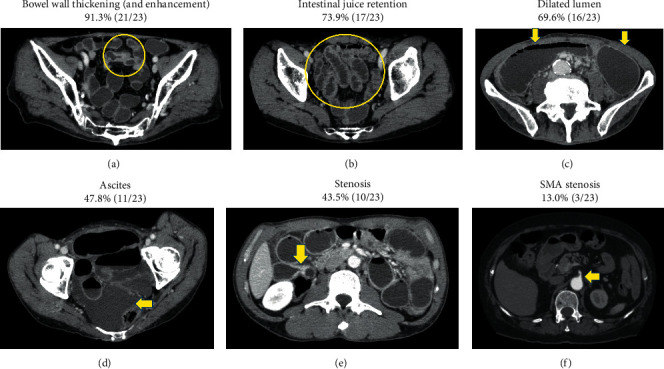
Positive computed tomography findings in patients with ischemic enteritis.

Twenty-three patients underwent CT scans, which included plain CT in 3 and contrast-enhanced CT in 20. Bowel wall thickening and intestinal juice retention were the most common findings, which were related to inflammation and stenosis at the lesion (Figure 1).

### 2.2. DBE

**Figure 2:**
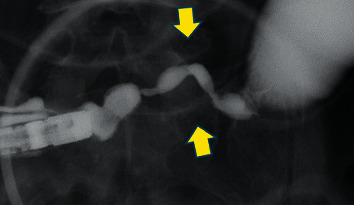
Contrast medium enterography. Arrow indicates the lesion site which was 4 cm in length.

All 24 patients underwent DBE, which involved peroral DBE alone (3 patients), transanal DBE alone (12 patients), and both peroral and transanal DBE (9 patients). We observed the entire small intestine using DBE, including enterography using contrast medium (Gastrografin ®) (Figure 2), in 10 patients (transient IE: 4 patients; stenotic IE: 6 patients). The median interval from symptom onset to DBE was 58 days (range: 7–154 days) for transient IE and 140 days (range: 16–630 days) for stenotic IE. There were no serious DBE-related adverse events, such as perforation, hemorrhage that required blood transfusions, or pancreatitis. Enterography using the contrast medium revealed that the stenotic lesion lengths were similar for both transient and stenotic IE, although stenotic IE cases had a significantly higher stenosis ratio (81% vs. 63%, *P*=0.033) (Table 3).

**Figure 3:**
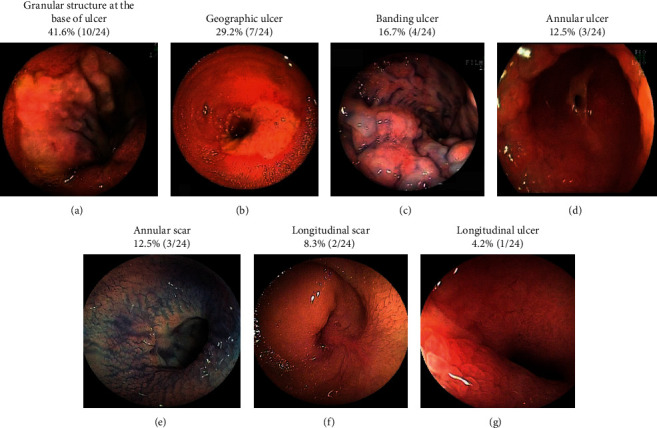
Endoscopic images from the stenosis site during the double-balloon endoscopy.

**Figure 4:**
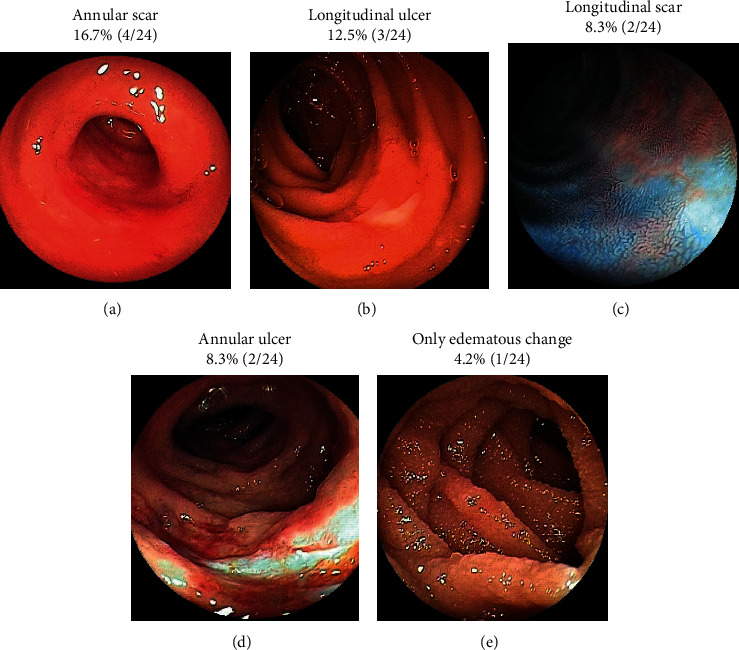
Endoscopic images in the small bowel, excluding the stenosis site, during double-balloon endoscopy.

Biopsy was performed for 18 patients who had ulceration at the edge of segmental stenosis in the small bowel. Three of 5 transient IE cases involved ischemic changes that were identified by the pathologist, although ischemic changes were only observed in 5 of 13 stenotic IE cases. Although the endoscopist usually obtained a biopsy after any positive findings during DBE, a biopsy specimen was not obtained in 6 patients. Among those 6 patients, biopsy was not possible in 4 patients who had been administered 2 types of anticoagulant agents; in the other 2 patients, excessive stenosis precluded a biopsy. A granular structure at the ulcer base was the most common DBE finding at the stenotic site (Figure 3), although other ulcer types were observed in the other segments of the small bowel (Figure 4).

### 2.3. Small Bowel Enteroclysis

**Figure 5:**
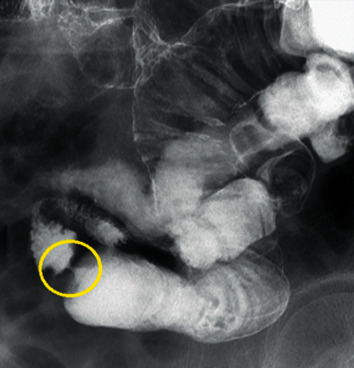
Small bowel enteroclysis. Stenosis was very severe, and the oral lumen was dilated.

**Figure 6:**
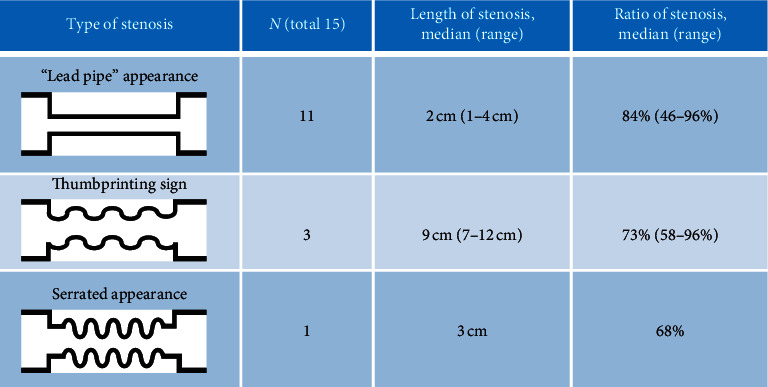
Types of positive findings at the stenosis site during small bowel enteroclysis.

Fifteen patients underwent small bowel enteroclysis (Figure 5), which revealed the “lead pipe” sign (11 patients), thumbprinting (3 patients), and the serrated lumen sign (1 patient). The group with the lead pipe sign had a shorter length of stenosis but a higher ratio of stenosis (Figure 6).

### 2.4. Treatments

One of the 9 patients with transient IE required an endoscopic hemostatic procedure, while the other 8 were treated conservatively (Table 3). None of the patients with transient IE experienced recurrence. The 15 patients with stenotic IE underwent surgical treatment (11 patients), endoscopic balloon dilation (3 patients), and observation (1 patient). Ischemic changes in the surgical specimen were histologically diagnosed in 5 of the 11 patients who underwent surgery. The patient who underwent observation also received central venous nutrition because of a very poor general condition and experienced recurrence at 31 days after discharge. The median interval from the onset of major symptoms to the operation was 157 days (range: 72–651 days), and the median interval from the onset of major symptoms to endoscopic balloon dilation was 201 days (range: 44–349 days).

## 3. Discussion

Ischemic bowel disease is a heterogenous group of disorders that includes acute and nonocclusive mesenteric ischemia, all of which involve hypoxia of the small intestine and/or colon [16]. However, while it can be difficult to distinguish between the specific disorders, it is important to clearly define IE, which involves a reversible ischemic disease without occlusion of a major artery. Although IE is rarer than ischemic colitis, the widespread use of balloon-assisted endoscopy has led to an increasing number of reports being published [17, 18]. To the best of our knowledge, no report has described the clinical features of IE, which makes it important to understand the patient characteristics, findings from various examinations (blood testing and external/endoscopic imaging), and treatment choices. Segmental stenosis on imaging and ischemic change in histopathological findings has an impact on the diagnosis of IE. Granular structures at the ulcer base would be specific to IE as they have not been reported in other types of enteritis. However, such findings are limited, being positive in less than 50% of cases. In other cases, differential diagnosis including biopsy specimen and the clinical course over more than 3 months is the key to confirming the diagnosis. Biopsy specimen can help in differentiating a small bowel tumor from IE. The clinical course would differentiate other kinds of infectious enteritis from IE, namely, intestinal Anisakis or worms. We did not detect any significant sex-based differences, although stenotic IE was significantly more common in male patients. Most patients with IE are >60 years old [19], which agrees with our finding of an average age of 66 years at examination. Furthermore, most IE cases are complicated by hypertension, diabetes mellitus, ischemic heart disease, or cerebral infarction, which agrees with the association between IE and atherosclerosis [8, 16]. A previous report has also indicated that the risk factors for IE include smoking, vasculitis, fibromuscular dysplasia, and arterial dissection [20].

The symptoms caused by small bowel stricture and melena may be a trigger for diagnosing IE, as well as the incidental identification of small bowel stenosis during imaging. Abdominal adhesions related to previous surgery are the most common cause of small bowel stenosis [21]. However, the diagnosis of IE should exclude adhesive intestinal obstruction, and differentiation based on laboratory markers of inflammation, as well as other imaging examinations, is needed. Nevertheless, laboratory test results are often unhelpful in the clinical setting [9]; however, we observed positive CT findings for 95.8% of our patients.

The most common CT finding was segmental bowel wall thickening, which may guide the diagnosis of IE, and is occasionally associated with the target sign on CT. The target sign is not typically associated with malignancy [22] and nonspecifically reflects the presence of a thickened bowel wall, which is typically related to inflammatory disease (including IE) [22]. Decreased segmental bowel wall enhancement is significantly associated with surgically identified small bowel ischemia [23], which can involve acute mesenteric ischemia and nonocclusive mesenteric ischemia. Thus, we believe that contrast-enhanced CT is essential for differentiating between IE and other diseases. The use of imaging should also be considered in the context of the patient's clinical background [24], as 3 patients had stenosis or dissociation of the superior mesenteric artery that was related to IE. Nevertheless, the lack of specific laboratory test parameters still complicates the diagnosis of IE [25].

IE cases are classified as transient or stenotic [5], although a previous report also classified IE as occlusive and nonocclusive [25]. We classified our patients as having transient or stenotic IE based on the endoscopic and contrast radiography findings. The literature on endoscopic findings of IE has been limited to case reports [8, 10, 26], although we also observed a diverse set of ulcers, including annular, longitudinal, banding, and geographic ulcers. In our patients, the clinical features of the two types of IE were similar; however, the stenotic type involved a significantly greater number of male and thinner patients and led to persistent stenotic symptoms. Stenotic IE cases had a significantly higher ratio of lesion stenosis. Transient IEs did not involve geographic ulcers connected to the stenosis except for two cases, although a granular pattern was often observed at the ulcer base, especially in geographic ulcers. One of the histological characteristics of IE is deep UL-II or UL-III ulcers [8, 27, 28], and UL-III ulcers typically become scars during the healing stage. We assume that these lesions involve acute and severe local inflammation, which subsequently converts to fibrosis during the healing process. A previous report has summarized the radiographic findings of IE [29], although we believe that ours is the first report to review the endoscopic findings. Our radiographic findings included afferent tubular changes, as previously reported [8, 28], and the IE-related stenosis was categorized as tubular, concentric, or edematous (Figure 6). These findings were observed in both transient and stenotic IE and may be a useful reference for diagnosing IE. Moreover, the stenotic length was similar between transient and stenotic IE, although stenotic IE was associated with a significantly higher ratio of stenosis. This characteristic may be useful for distinguishing between transient and stenotic IE and in predicting the prognosis of the stenotic lesion.

Because there are no established diagnostic criteria for IE, endoscopic findings are crucial and the histological findings also typically play a decisive role. The histological characteristics of IE include [8, 28] variable ulcer depths (typically UL-II or UL-III), an ulcer base lined with vessel-rich granulation tissue, severe fibrosis mainly observed within the submucosal layers, severe inflammatory cell invasion (primarily lymphocytes and plasma cells), and hemosiderin-laden macrophages scattered throughout the entire thickness of the intestine. In fact, ischemic changes were only observed in 5 of 13 cases of stenotic IE (38.4%). This suggests that the lumen was narrower in stenotic IE (Table 3), and a good sample was not completely obtained.

It is also important to distinguish IE from other diseases. For example, the differential diagnosis of transient IE should also consider infectious enteritis and intestinal anisakiasis. In this context, the patient's general condition will improve over several days, and the diagnosis of intestinal anisakiasis can be guided by a history of eating raw fish and serum positivity for anisakiasis antibodies. The differential diagnosis of stenotic IE should also consider Crohn's disease, intestinal tuberculosis, nonsteroidal anti-inflammatory drug-related ulcers, chronic nonspecific multiple ulcers of the small intestine, radiation enteritis, and malignant tumors, such as small bowel carcinoma and malignant lymphoma. A clinical history of medication and radiation, as well as blood test results of interferon-*γ* for tuberculosis, are useful for reaching definitive diagnosis. For example, patients with Crohn's disease present with full-thickness inflammation of the intestinal wall, and small bowel obstruction will occur through either acute inflammation leading to occlusion or chronic inflammation leading to strictures [3]. Malignant lesions are also likely to have some symptomatic presentation [3].

We did not observe recurrence in any case that involved transient IE, and the one patient who experienced recurrence of stenotic IE had a very poor general condition. One report has indicated that patients avoided surgery when the stricture length was <3 cm [7], and all our cases that involved endoscopic balloon dilation also had a stenotic length <3 cm. Nevertheless, many cases that involved surgery also had a stenotic length <3 cm. Given the possibility of stenosis co-occurring with ulcers or the possibility of multiple stenoses, the treatment strategy for IE must be carefully and rationally selected. We have also proposed new diagnostic criteria for IE based on a previous report and our findings from this study (Table 4). Conventional criteria used the phrase “clinical and radiographical findings suggestive of ischemic enteritis,” which was unclear because the macroscopical details of the lesions were not understood. In this study, we detected wall thickness in 87.5% of patients on imaging (21/24 patients) on imaging and ulceration at the edge of the stenosis in 75.0% (18/24 patients) by DBE. Therefore, we included these 2 factors in the diagnostic criteria to clarify the process of identification of clinical and radiographical findings. Possible ulceration in the segmental stenotic area should be evaluated using small bowel endoscopy, small bowel barium study, or alternative strategies, based on the possibility of inflammation leading to ulceration and subsequent cannular stenosis. Currently, DBE has the ability to detect the ulceration at the edge of the stenosis in any site of the small bowel.

This study has some limitations, which include the small single-center retrospective design. Furthermore, there is no gold-standard technique for clinically diagnosing IE. IE exhibits several types of ulcerations, and there are no consistent endoscopic findings on DBE, although there might be some tendencies. The new diagnostic criteria have a limitation in terms of DBE findings. We divided IE patients into two types, “transient” and “stenotic,” according to the concept of ischemic colitis. The difference between the two types of IEs is that they may be affected by the severity of ischemia in the small bowel wall. However, the severity of ischemia may decline and change over time. Therefore, we were not able to confirm the ischemic change at the time of histopathological examination in some patients. To elucidate these changes, further verification based on the results of original articles and reviews is required.

The severity of ischemia in the small bowel wall can affect the macroscopic features of IE. There were 6 patients who did not have ischemic change in the surgical specimens over the course of the IE, thus exhibiting the possibility of recanalization of the small bowel vessel. In this study, such patients were clinically diagnosed with IE according to clinical and radiographical criteria suggestive of IE, based on time-dependent changes and excluding other diagnoses. However, we hope that our findings will be helpful for guiding the development of a diagnostic strategy for IE.

In conclusion, our IE cases had endoscopic findings that typically involved cannular stenosis with extended ulceration spreading over the edge of stenosis, as well as a granular appearance at the ulcer base. These findings may be helpful in guiding the diagnosis of IE.

## Figures and Tables

**Table 1:** Conventional diagnostic criteria.

Diagnosis of ischemic enteritis fulfills criteria 1 and 3 or criteria 2 and 3
1. Histopathological findings from a endoscopic biopsy specimen or resected bowel segment compatible with ischemic enteritis.
2. Clinical and radiographical findings suggestive of ischemic enteritis based on time-dependent changes.
3. Exclusion of other known etiologies, such as Crohn's disease, infectious disease (intestinal tuberculosis, intestinal anisakiasis, and others), NSAID-related ulcers, chronic nonspecific multiple ulcers of the small intestine, radiation enteritis, or malignant tumor based on clinical course and histopathological findings. NSAID, nonsteroidal anti-inflammatory drug.

**Table 2:** Patient characteristics.

Ischemic enteritis		*n* = 24
Clinical		
Sex, male/female	—	14/10
Age in years, median (range)	—	66 (36–86)
Body mass index, mean ± SD	—	21.0 ± 4.1
Surgical history, *n*	—	7
Underlying disease, *n* (duplicated)	Cerebrovascular and cardiovascular disease	12
	Hypertension	11
	Diabetes	6
	Dyslipidemia	5

Symptom, *n*	Abdominal pain	19
	Nausea or vomiting	7
	Melena	5

Lesions		
Type, transient/stenosis		9/15
Stenotic symptoms at admission		19

**Table 3:** Comparing transient and stenotic ischemic enteritis based on patients' characteristics.

	Transient type	Stenosis type	*P* value
*N*	9	15
Age, years old, median (range)	64 (36–83)	68 (45–86)	0.339
Sex, male/female	2/7	12/3	<0.001
Body mass index, mean±SD^ǂ^	22.5 ± 5.0	20.1 ± 3.2	0.143
Surgical history, *n*	2	5	0.668

Underlying disease, *n* (duplicated)			
Cerebrovascular and cardiovascular disease	4	8	0.999
Hypertension	3	8	0.422
Diabetes	3	3	0.634
Dyslipidemia	2	3	0.999

Symptoms, *n* (duplicated)			
Abdominal pain	6	13	0.325
Nausea or vomiting	2	5	0.668
Melena	3	2	0.325
Stenotic symptoms at admission	4	15	0.006

Lesions			
Single/multiple	7/2	9/6	0.657
Jejunum/ileum	2/7	6/9	0.657
Stenosis, *n*	4	15	0.006

Contrast medium enterography			
Length of stenosis, cm, median (range)	5 (2–10)	6 (2–10)	0.759
Ratio of stenosis, %, median (range)	63 (43–77)	81 (46–96)	0.033

Laboratory data at admission			
Total protein (g/dL), mean ± SD	6.3 ± 1.4	6.0 ± 1.0	0.384
Albumin (g/dL), mean ± SD	3.4 ± 1.1	2.9 ± 1.0	0.315
CRP (mg/dL), mean ± SD	3.3 ± 6.6	4.0 ± 3.7	0.216
White blood cells (×103/*μ*L), mean ± SD	8.6 ± 4.2	7.1 ± 2.8	0.547
Hemoglobin (g/dL), mean ± SD	10.3 ± 2.7	11.2 ± 2.5	0.367
Platelet (×104/*μ*L), mean ± SD	26.1 ± 15.3	30.1 ± 8.5	0.171

CT findings, *n*	8	15	—
Stenosis of superior mesenteric artery	1	2	0.999
Bowel wall thickening	7	14	0.999
Dilated lumen	5	11	0.657
Intestinal juice retention	7	10	0.369
Stenosis	5	5	0.221
Ascites	5	6	0.400

Endoscopic images from the stenosis site, *n*	9	15	—
Annular ulcer	1	2	0.999
Longitudinal ulcer	1	0	0.375
Banding ulcer	2	2	0.999
Geographic ulcer	1	6	0.190
Annular scar	1	2	0.999
Longitudinal scar	1	1	0.999
Granular structure at the base of ulcer	2	8	0.209

Endoscopic images from the nonstenosis site, *n*	9	15	—
Annular ulcer	1	1	0.999
Longitudinal ulcer	1	2	0.999
Annular scar	2	2	0.999
Longitudinal scar	1	1	0.999
Only edematous change	1	0	0.375

Small bowel enteroclysis, *n*	4	11	—
Leadpipe appearance	2	9	0.516
Thumbprinting sign	1	2	0.999
Serrated appearance	1	0	0.266

Taking biopsy, *n*	5	13	—
Ischemic change	3	5	0.607

Treatment			
—	—	—	—
—	Hemostasis: 1	Surgical resection: 11	—
—	Observation: 8	EBD^*∗*^: 3	—
—	—	Observation: 1	—

Observation period, days, median (range)	459 (174–1616)	878 (24–2332)	0.114
Recurrence, *n*	0	1	—

^*∗*^Endoscopic balloon dilation. ^ǂ^Standard deviation.

**Table 4:** New diagnostic criteria for ischemic enteritis.

Diagnosis of ischemic enteritis fulfilling criteria 1 and 3 or criteria 2 and 3
(1) Histopathological findings from an endoscopic biopsy specimen or resected bowel segment compatible with ischemic enteritis
(2) Segmental wall thickness on imaging or ulceration^*∗*^ on endoscopic images, suggestive of ischemic enteritis based on time-dependent changes
(3) Exclusion of other known etiologies, such as Crohn's disease, infectious disease (intestinal tuberculosis, intestinal anisakiasis, and others), NSAID-related ulcers (chronic nonspecific multiple tuberculosis, intestinal anisakiasis, and others), NSAID-related ulcers, chronic nonspecific multiple ulcers of the small intestine, radiation enteritis, or malignant tumor based on clinical course and histopathological findings

^*∗*^Some types of ulceration and granular structures at the ulcer base.

## Data Availability

The data used to support the findings of this study are available from the corresponding author upon request.

## Abbreviations

DBE: Double-balloon endoscopy
IE: Ischemic enteritis
CT: Computed tomography.
